# A Nine-Year Review of *Acinetobacter baumannii* Infections Frequency and Antimicrobial Resistance in a Single-Center Study in Salerno, Italy

**DOI:** 10.3390/pathogens14111165

**Published:** 2025-11-14

**Authors:** Enrica Serretiello, Mariagrazia De Prisco, Giuseppe Di Siervi, Ilaria Cosimato, Federica Dell’Annunziata, Emanuela Santoro, Emilia Anna Vozzella, Giovanni Boccia, Veronica Folliero, Gianluigi Franci

**Affiliations:** 1 U.O.S. Microbiology and Virology, AOU San Giovanni di Dio e Ruggi d’Aragona, 84131 Salerno, Italy; enrica.serretiello@unicampania.it (E.S.); gfranci@unisa.it (G.F.); 2 Department of Medicine, Surgery and Dentistry “Scuola Medica Salernitana”, University of Salerno, Baronissi, 84081 Salerno, Italy; depriscomariagrazia22@gmail.com (M.D.P.); ilaricos96@gmail.com (I.C.); esantoro@unisa.it (E.S.); gboccia@unisa.it (G.B.); 3 Postgraduate School in Clinical Pathology and Clinical Biochemistry, University of Salerno, Baronissi, 84081 Salerno, Italy; giuseppedisiervi200@gmail.com; 4 University Hospital “San Giovanni di Dio e Ruggi d’Aragona”, 84121 Salerno, Italy; direzione.sanitaria@sangiovannieruggi.it; 5 UOC Hospital and Epidemiological Hygiene, San Giovanni di Dio and Ruggi D’Aragona University Hospital, 84131 Salerno, Italy

**Keywords:** antimicrobial resistance, ESKAPE, polymicrobial infections, nosocomial infections, retrospective study, COVID-19

## Abstract

*Acinetobacter baumanni* (*A. baumannii*) is a well-known pathogen associated with antimicrobial-resistant infections. It is a major cause of nosocomial infections and is frequently associated with polymicrobial and antibiotic-resistant infections. This study investigates the frequency of *A. baumannii* infections, its antimicrobial resistance profile and the main co-pathogens isolated in respiratory samples at the San Giovanni di Dio e Ruggi d’Aragona Hospital in 2015–2019 (pre-COVID-19 pandemic) and 2020–2023 (during/post-COVID-19 pandemic). Bacterial identification and antibiotic susceptibility testing were performed using the VITEK^®^ 2 system (2015–2019), while identification was carried out with MALDI-TOF MS starting from 2020. A total of 1679 strains were isolated between 2015 and 2019, and 1186 between 2020 and 2023, with significantly higher frequencies in males 61–80 and females 71–80. *A. baumannii* was isolated predominantly from respiratory specimens, derived predominantly in intensive care units (ICUs). The antimicrobial resistance rates of *A. baumannii* were above 90% for gentamicin, trimethoprim/sulfamethoxazole, imipenem and ciprofloxacin, while colistin resistance was less than 1% (0.95%) in pre-pandemic and alarmingly increased during/post pandemic period (6.1%)*. A. baumannii* was most frequently associated with *Klebsiella pneumoniae*, *Staphylococcus aureus* and *Pseudomonas aeruginosa* in respiratory tract infections. *A. baumannii* represents a serious global health threat due to its extensive antimicrobial resistance, highlighting the need for continuous surveillance, detailed strain characterization, and development of new antimicrobial agents.

## 1. Introduction

*Acinetobacter baumannii* (*A. baumannii*), a member of the ESKAPE group (*Enterococcus* spp., *Staphylococcus aureus* (*S. aureus*), *Klebsiella pneumoniae* (*K. pneumoniae*), *A. baumannii*, *Pseudomonas aeruginosa* (*P. aeruginosa*), *Enterobacter* spp. group), is considered a major concern by the World Health Organization (WHO) [1]. It is recognized as a major pathogen responsible for nosocomial infections, such as ventilator-associated pneumonia (VAP), central line-associated bloodstream infections (CRBSI), urinary tract infections (UTIs) and surgical site infections. Its ability to survive and proliferate on both dry and moist surfaces, including hospital equipment, leads to particularly daunting challenges in controlling its nosocomial transmission in healthcare settings. Risk factors for *A. baumannii* infection include advanced age, severe underlying diseases, immunosuppression, significant trauma or burns, invasive surgery, and prolonged hospitalization. Multidrug-resistant (MDR) *A. baumannii* poses a serious public health threat due to the extremely limited therapeutic options. WHO has classified carbapenem-resistant *A. baumannii* (CRAB) as a critical-priority pathogen [2].

This antimicrobial resistance is particularly widespread, as confirmed by annual reports on antimicrobial resistance (AMR) published by the European Centre for Disease Prevention and Control (ECDC). Specifically, in 2015, antimicrobial resistance rates exceeding 80% were reported in several European countries, including Italy, Croatia, Greece, Cyprus, Lithuania, and Romania [3]. The resistance mechanisms of *A. baumannii* are multifaceted. For carbapenem resistance, more than 400 OXA-type β-lactamases have been identified [4]. The first enzyme identified in *A. baumannii* strains was OXA-23, which hydrolyzes antimicrobials such as ticarcillin, meropenem, amoxicillin, and imipenem. The OXA-40 enzymes, the second group recognized, confer antimicrobial resistance to penicillins but exhibit limited hydrolytic activity against cephalosporins and carbapenems. Additionally, other enzymes, such as OXA-24, OXA-58, and OXA-51, have also been described [5]. Carbapenem resistance in *A. baumannii* is not only mediated by carbapenemases but can also result from the loss or downregulation of porins such as OmpA and CarO. In addition to carbapenem resistance, other resistance mechanisms contribute to antimicrobial resistance across different antibiotic classes. Mutations in the *gyrA* and *parC* genes can confer antimicrobial resistance to quinolones, while reduced membrane permeability can limit the effectiveness of β-lactams, aminoglycosides, and tigecycline. Furthermore, modifications in lipopolysaccharides (LPS) can induce resistance to polymyxins [6]. In 2015, fluoroquinolone resistance in *A. baumannii* exceeded 50% in 15 European countries. Similarly, aminoglycoside resistance rates were above 50% in 10 countries, including Italy. More concerning is the combined antimicrobial resistance to carbapenems, quinolones, and aminoglycosides, which was detected in 43.6% of isolates in 2019 [7]. Since the first case of colistin resistance was reported in the Czech Republic in 1999, its frequency of *A. baumannii* strains has increased annually [8,9]. Due to the high frequency of MDR *A. baumannii*, maintaining the effectiveness of available antibiotics requires ongoing local surveillance to monitor emerging resistance and guide rational antibiotic use.

In this context, this study aimed to evaluate the frequency and patterns of antimicrobial resistance in *A. baumannii* isolates collected from hospitalized patients at San Giovanni di Dio e Ruggi d’Aragona Hospital (Salerno, Italy) between 2015 and 2023. The study also sought to explore temporal differences between the pre-COVID-19 and post-COVID-19 periods to better understand potential changes in infection dynamics and resistance trends within the hospital setting.

## 2. Materials and Methods

### 2.1. Data Collection

Data collected between 2015 and 2023 at the Microbiology Laboratory of San Giovanni di Dio e Ruggi d’Aragona Hospital (Salerno, Italy) were retrieved and analyzed from the laboratory database. The San Giovanni di Dio and Ruggi d’Aragona University Hospital is a tertiary-level referral and training center, serving as a leading hub for advanced medical care and research in Southern Italy. The hospital has a capacity of over 700 beds and includes a wide range of specialized units, such as intensive care, surgery, medicine, and infectious diseases, providing comprehensive diagnostic and therapeutic services to both inpatients and outpatients throughout the Campania region. All patients included in the study were hospitalized within the hospital complex. Demographic information, including age, sex, department of admission, and infection site, was recorded for each patient included in the study. Patients were classified by gender and stratified into age groups (0–10, 11–20, 21–30, 31–40, 41–50, 51–60, 61–70, 71–80, 81–90, 91–100 years). This information was used to evaluate the distribution of A. baumannii infections across different patient groups and hospital departments, as well as to identify trends in susceptibility and antimicrobial resistance patterns within the patient population.

### 2.2. Biological Samples Collection and Processing

*A. baumannii* isolates were isolated from respiratory samples (sputum, bronchoaspirate, and bronchoalveolar lavage), wound swabs, blood cultures, urine, catheter and cerebrospinal fluid cultures, vaginal swabs, and other clinical specimens. Clinical specimens were processed according to standard clinical microbiology guidelines. Blood samples were inoculated into blood culture bottles and incubated in the BD BACTEC™ 9240Automated Blood Culture Monitoring System (Becton Dickinson Diagnostic Instrument Systems, Sparks, MD, USA) for up to 5 days. In case of positivity, a drop of emoculture was plated onto chocolate agar (incubated under 5% CO_2_ for 24–48 h), tryptic soy agar, Columbia naladixic acid agar, McConkey, and Sabouraud glucose agar culture media (bioMérieux-l’Etoile, Marcy-l’Étoile, France, observed at 24 h and 48 h after incubation. Respiratory tract specimens, wound swabs, and other specimens were plated directly onto the same standard culture medium as above. Catheters were placed in liquid enrichment medium for the first day, in case of cloudy medium, subsequently were plated on the above-mentioned medium. Urine samples were plated on CHROMID^®^ CPS^®^ Elite (CPSE) chromogenic plates (bioMérieux-l’Etoile, Marcy-l’Étoile, France). All plates and liquid media were incubated at 37 °C for 24–48 h.

### 2.3. Bacterial Identification and Antimicrobial Profile

Bacterial identification and antimicrobial susceptibility testing were performed using the VITEK2 system (bioMérieux, Marcy l’Etoile, France) and from 2021 the identification was implemented by the MALDI-TOF (Matrix Assisted Laser Desorption Ionization-Time of Flight) MS (bioMérieux, Marcy l’Etoile, France) technology, according to the manufacturer’s guidelines. Pure bacterial colonies were suspended in a tube containing 3 mL of 0.45% sodium chloride solution and the bacterial suspension was adjusted to a McFarland standard of 0.5 using Densichek (bioMérieux, Marcy l’Etoile, France). The ID-GN identification card was used for bacterial identification, while the AST-379, AST-397, and subsequently implemented by AST-438 cards were used for antimicrobial susceptibility testing. The tested antimicrobials were Ciprofloxacin, Colistin, Imipenem, Trimetoprimim/Sulfaetaxole, Gentamicin, and Amoxicillin-cavulanic acid. Reported colistin resistance was determined by the broth microdilution method. Antimicrobial susceptibility results were interpreted according to EUCAST guidelines (https://www.eucast.org/ (accessed on 12 January 2024). Intermediate (I) isolates were excluded from the analysis to maintain methodological consistency and avoid ambiguity in the classification of antimicrobial susceptibility. Since the “susceptible, increased exposure” category does not necessarily reflect resistance or clinical susceptibility, its inclusion could have introduced variability and potential bias in the trend analysis. Therefore, only the “susceptible (S)” and “resistant (R)” categories were considered to ensure robust and comparable results across years.

### 2.4. Data and Statistical Analysis

Statistical analyses were performed using R software (version 12.1.4). Before analysis, a deduplication procedure was applied at the patient/episode level to minimize bias from multiple isolates obtained from the same patient. Only one isolate per patient per infection episode (defined as a 14-day interval from the first positive result) was included. When multiple samples were collected on the same day, the isolation from a sterile site (blood) or, if unavailable, the most clinically relevant specimen was selected. The chi-square test was applied to assess a significant difference in the distribution of antimicrobial resistance and susceptibility during the study years. Additionally, Fisher’s exact test was performed to analyze correlations between gender and age groups in data with uneven distribution. The Cochrane-Armitage test was used to assess the statistical significance of antimicrobial resistance trends for each antibiotic test. For all statistical tests, the confidence level was set at 5%. A logistic regression was applied from 2015 to 2023, used to assess the trend in antimicrobial resistance over time, since the response variable (resistant or susceptible) is binary and to estimate the change in the log-odds of antimicrobial resistance for each year, thus quantifying whether the probability of antimicrobial resistance increases or decreases over time. The odds ratio indicates the extent to which the relative likelihood of developing antimicrobial resistance changes from one year to the next. A *p*-value was calculated to determine whether the observed antimicrobial resistance trend was statistically significant. To account for multiple simultaneous comparisons across different antibiotics, the Benjamini–Hochberg correction was applied to control the false discovery rate (FDR), representing the expected proportion of false positives among tests declared significant. This approach is appropriate when analyzing multiple independent or moderately correlated tests and ensures that reported trends reflect robust associations rather than spurious findings.

## 3. Results

### 3.1. Frequency of A. baumannii Strains and Patients’ Gender Distribution

A total of 1679 *A. baumannii* strains were isolated from patient samples collected between January 2015 to December 2019 and 1186 between January 2020 to December 2023 at the University Hospital “San Giovanni di Dio e Ruggi d’Aragona” in Salerno (Italy). A higher number of *A. baumannii*-positive infections was observed among male patients in both periods. During 2015–2019, 65.3% of infections occurred in males and 34.7% in females. In the 2020–2023 period, the distribution remained similar, with 64.4% of infections in males and 35.6% in females (Table 1).

### 3.2. Gender Distribution of Positive A.baumannii Patients Stratified by Age Group

*A. baumannii*-positive patients were stratified by age group. Stratification revealed that the highest percentage of *A. baumannii* infection occurred in the 61–70 age group in 2025–2019 and 71–80 in 2020–2023 for males and 71–80 age group for females in both periods (Table 2 and Table 3).

### 3.3. Frequency of A. baumannii Strains by Biological Matrices and Infection Sites

The distribution of *A. baumannii* across several biological matrices over the nine years of investigation was also evaluated. Predominantly, *A. baumannii* was found in respiratory samples, accounting for 46.6% of the isolates, followed by wound swab samples (14.8%), blood culture (11.7%), and urinary samples (10.9%) in 2015–2019. In 2020–2023, the highest rate of infection in the respiratory tract was confirmed (45.2). Blood culture (15%), urine culture (14.4%) and cultural swab (10.7%) resulted differently stratified in contrast to the pre-pandemic period. Below a 10% frequency, *A. baumannii* was found to be less widely isolated from other specimen types, including cultural swabs, catheter liquor cultures, vaginal swabs, and other samples. In Table 4 and Table 5 the detailed distribution of *A. baumannii* isolates is reported.

### 3.4. A. baumannii Frequency Across Different Hospital Units

**Figure 1:**
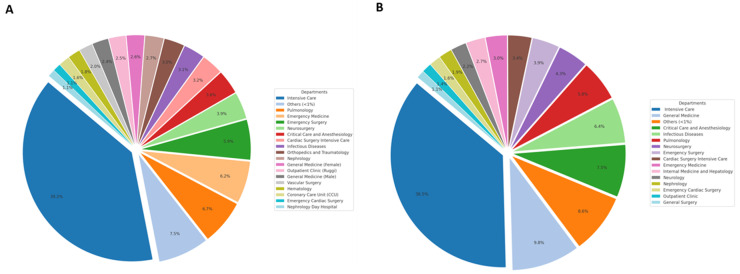
Distribution of *A. baumannii* isolates among hospital units in 2015–2019 (**A**) and 2020–2023 (**B**). The figure illustrates the distribution of *A. baumannii* isolates across various hospital units during the two study periods. “Other” refers to units with a frequency less than 1%, as detailed in Supplementary Table S1.

A stratification of *A. baumannii* infections by hospital unit was performed. As shown in Figure 1, in the Intensive Care Unit (ICU), it resulted in the highest frequency of *A. baumannii* positive samples, accounting for 39.2% in the 2015–2019 period and 36.5% in 2020–2023 (Figure 1). Although the rate of isolates found in patients with access to the different departments is different between the two periods, in general, *A. baumannii* has been mostly reported in Pulmonology, Emergency Medicine, General Medicine, Critical Care, and Anesthesiology. In others, all departments showing a frequency <1% were included (Supplementary Table S1).

### 3.5. Antimicrobial Resistance Profile of A. baumannii and Its Temporal Trends

**Figure 2:**
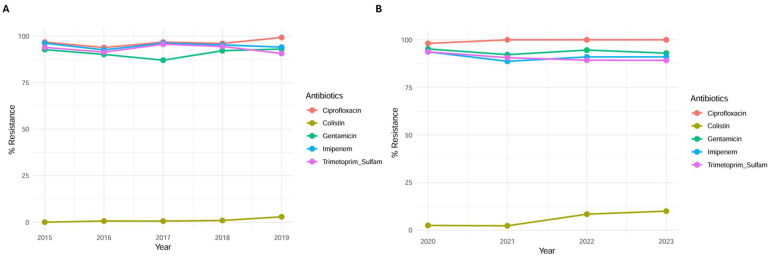
Antimicrobial resistance of *A. baumannii* over time: (**A**) 2015–2019 and (**B**) 2020–2023. The figure illustrates the temporal trends in antibiotic resistance rates of *A. baumannii* isolates across the two study periods. Each panel shows the percentage of resistant isolates for the tested antibiotics, highlighting variations and emerging resistance patterns over time.

Antimicrobial resistance patterns of *A. baumannii* isolates were analyzed across various antibiotics, with a particular focus on identifying significant trends over time. An important and substantial increase in antimicrobial resistance was observed for ciprofloxacin, rising from 96.85% in 2015 to 99.31% in 2019 (*p*-value trend = 0.04), and for colistin, which increased from 0% in 2015 to 2.9% in 2019 (*p*-value trend < 0.001). During the/post-COVID-19 period, ciprofloxacin resistance further increased significantly, from 98.1% in 2021 to 100% in 2023 (*p*-value trend 0.001). Similarly, colistin resistance showed a marked rise from 2.5% in 2021 to 10% in 2023 (*p*-value trend < 0.001). Conversely, gentamicin resistance exhibited a significant decline, decreasing from 93.7% in 2015 to 90.6% in 2019 (*p*-value trend 0.05). As illustrated in Figure 2, overall antimicrobial resistance patterns among *A. baumannii* isolates remained extremely high throughout both study periods (2015–2019 and 2020–2023). In both periods, ciprofloxacin, imipenem, and trimethoprim/sulfamethoxazole showed consistently high resistance rates (>90%), indicating a sustained multidrug resistance profile of *A. baumannii*. Gentamicin showed moderate levels of resistance with little variation from year to year. In contrast, colistin maintained the lowest resistance rates, although a slight upward trend was observed in the second period (2020–2023).

### 3.6. Regression Analysis of A. baumannii Antimicrobial Resistance Profile for Each Year Investigated

**Figure 3:**
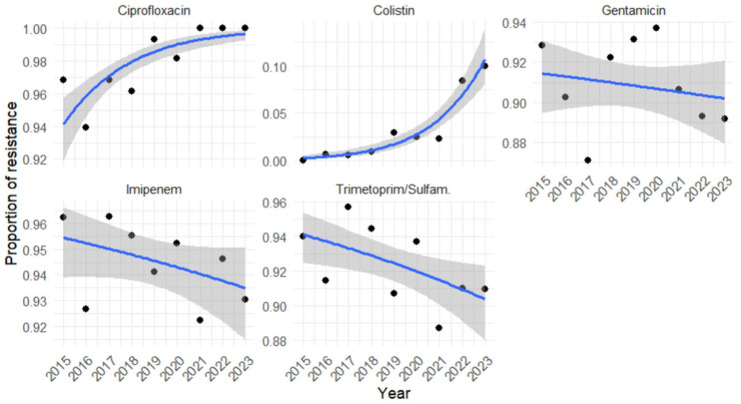
Logistic regression analysis of *A. baumannii* antimicrobial resistance trends over time (2015–2023). The figure shows the results of the logistic regression analysis evaluating the temporal trend of antibiotic resistance in *A. baumannii* isolates from 2015 to 2023. The blue line represents the predicted proportion of resistance over time according to the logistic regression model, while the gray shaded area represents the 95% confidence interval (CI) around the prediction. For each antibiotic, the odds ratio (OR) with the corresponding 95% CI and *p*-value is reported. Statistically significant changes (*p* < 0.05) are highlighted.

Antimicrobial resistance trends over time were assessed using regression models applied to annual data, using the binary outcome (resistant or susceptible) as the response variable Logistic regression analysis of *A. baumannii* antimicrobial resistance trends from 2015 to 2023 revealed distinct temporal patterns across antibiotics (Figure 3, Table 6). *A. baumannii* isolates showed a significant increase in resistance to ciprofloxacin over time (OR = 1.44, 95% CI 1.27–1.63, *p* < 0.001), with the odds of resistance rising by approximately 44% each year. Colistin demonstrated an even stronger and highly significant upward trend (OR = 1.61, 95% CI 1.43–1.82, *p* < 0.001), corresponding to an estimated 61% annual increase in resistance odds, underscoring a concerning escalation in resistance to one of the last-resort agents. In contrast, gentamicin (OR = 0.98, 95% CI 0.93–1.03, *p* = 0.45) and imipenem (OR = 0.95, 95% CI 0.90–1.01, *p* = 0.13) exhibited no statistically significant changes over time, suggesting relative stability in resistance patterns despite persistently high frequency levels. Finally, trimethoprim/sulfamethoxazole (OR = 0.94, 95% CI 0.89–0.98, *p* = 0.01) was the only antibiotic showing a significant downward trend, with an approximate 6% annual reduction in the odds of resistance. Although this decline is modest, it may indicate the beginning of a favorable shift in susceptibility that warrants further monitoring.

### 3.7. A. baumannii Coinfections in Respiratory Matrices

A stratification of *A. baumannii* co-infection in respiratory samples was performed. The co-pathogen species with the highest frequency was *K. pneumoniae* (28.8%), followed by *S. aureus* (22.2%) and *P. aeruginosa* (14.8%) in 2015–2019. Differently in 2020–2023, we found that *A. baumannii* most frequently co-occurred with *S. aureus* (28.6%), *P. aeruginosa* (18.4%), and *K. pneumoniae* (15.0%). The remaining bacterial isolates were found at a percentage <10%.

## 4. Discussion

*A. baumannii* is a multidrug-resistant opportunistic pathogen responsible for severe healthcare-associated infections, including ventilator-associated pneumonia, sepsis, wound infections, post-surgical meningitis, and urinary tract infections, particularly in immunocompromised or long-term hospitalized patients [10]. Given the increasing global concern over this MDR organism, the present study investigated the frequency and distribution of *A. baumannii* infections in our region. To account for shifts in hospital admission policies, empirical therapy protocols, and the widespread use of mechanical ventilation and intensive care during the COVID-19 pandemic, data were analyzed separately for the pre-pandemic (2015–2019) and pandemic/post-pandemic (2020–2023) periods [11]. The results are presented descriptively, acknowledging differences in population structure and hospital dynamics between the two intervals. As highlighted by the European Centre for Disease Prevention and Control, infection control measures implemented during the pandemic have influenced the epidemiology of several bacterial species [12,13].

We reported data on the frequency of *A. baumannii* in our Hospital (San Giovanni di Dio e Ruggi d’Aragona in Salerno) from January 2015 to December 2023. A total of 1679 isolates were identified during the pre-COVID-19 period (2015–2019) and 1186 during the COVID-19/post-COVID-19 period (2020–2023) (Table 1). The annual number of isolates varied, peaking in 2018 and reaching its lowest value in 2021. Overall, infections were more frequent in males (≈65%) than in females (≈34%), with the highest frequency among males aged 61–70 years and females aged 71–80 years (Table 2 and Table 3). The high frequency of *A. baumannii* infections in this age group seems to reflect a combination of factors related to hospitalization, immune vulnerability, and the demographic distribution of the hospitalized population [14]. Men generally have a lower life expectancy than women, so in older age groups, the number of women is higher [15]. Additionally, men aged 61–70 may be more exposed to infections due to pre-existing conditions such as cardiovascular or respiratory diseases, which increase the predisposition to nosocomial infections [16]. On the other hand, women aged 71–80 may be more susceptible to infections due to weaker immune systems and a higher hospitalization rate in advanced age. In support of our data, Tadese et al. indicated that the age of patients most exposed to these infections was 62 years [17].

In our study, *A. baumannii* was most frequently isolated from respiratory samples for both periods, followed by wound swabs (14.8%), blood cultures (11.7%), and urine cultures (10.9%) in 2015–2019 (Figure 1A). In 2020–2023, only a slight variation in the distribution of *A. baumannii* was recorded in the various districts, in which blood culture (15.3%) and urinary (13.6%) were higher than wound swab (10.8%) (Figure 2B). A U.S.A. study covering five years (2014–2019) analyzed the distribution of CRAB-mediated infections, reporting that 35.7% involved wound and soft tissue infections, followed by 32.1% respiratory tract infections. Consistent with our findings, these sites are among the most frequently affected. The highest mortality rates were reported for bloodstream infections (40.9%) and respiratory tract infections (21.9%) [18]. The increased use of broad-spectrum antimicrobials in several hospital wards, particularly in ICUs, may contribute to the selection of *A. baumannii* resistant strains. Indeed, in our hospital, the ICU covered the highest number of infection cases (more than 35%), followed by general medicine, critical care, anesthesiology, pneumology, emergency medicine, and emergency surgery, with a relatively constant distribution over the years. Many of these patients require medical devices during hospitalization, which are prone to bacterial biofilm formation, increasing the risk of device-related infections but can also exacerbate the underlying condition and promote the emergence of additional infectious strains [19]. Similarly, in the study conducted by Pogue et al., it was highlighted that (CRAB) is associated with higher mortality rates, prolonged hospital stays, and increased ICU admissions. Notably, 45.3% of patients with CRAB had their first positive sample collected during their ICU stay, emphasizing the critical role of intensive care units as a primary site for infection acquisition and spread. According to available data, ICUs are the hospital wards with the highest antimicrobial resistance levels, increasing the risk for vulnerable patients [18].

In our study, the antimicrobial resistance profile analysis showed that *A. baumannii* exhibits high antimicrobial resistance to most tested antimicrobials (Table 7 and Table 8). *A. baumannii* isolates showed a significant increasing trend in resistance to ciprofloxacin in both periods, while resistance to colistin rose from 0% in 2015 to 2.9% in 2019 and further to 10% in 2023. This increase, also highlighted by statistical trend analysis, reflects the pathogen’s progressive adaptation to the selective pressure exerted by the clinical use of colistin, one of the last antibiotics effective against multidrug-resistant strains. This phenomenon is particularly concerning in high-risk departments, such as intensive care and emergency medicine, where *A. baumannii* is frequently isolated and treatment options are already extremely limited. From a recent meta-analysis [20], colistin resistance is strongly associated with regional differences and a global increase appeared from 2012 to 2023 of about 5% [20]. Regarding carbapenems, *A. baumannii* showed a resistance rate of approximately 94% to imipenem, which remained stable over time and was consistent with national averages. Trimethoprim/Sulfamethoxazole and gentamicin showed an antimicrobial resistance rate of more than 90% in both periods, with an increase of more than 90%, like fluoroquinolones and carbapenems, which exhibited a fluctuating trend over time but without statistically significant variations.

A different trend from Italian and European data was highlighted by a 12-year retrospective analysis (2010–2021). This study observed a decline in antimicrobial resistance from 2010 to 2021, with a significant reduction in resistance to imipenem, meropenem, and amikacin, and low antimicrobial resistance to colistin (maximum 4% in 2018) and tigecycline (maximum 0.2% in 2019 and 2021). This suggests a decrease in MDR and possibly extensively drug-resistant (XDR) isolates, which were reduced by nearly half between 2010 and 2021. This trend is likely due to improvements in infection control, antimicrobial surveillance programs, and stricter antimicrobial stewardship in these countries [21]. These findings contrast with our study, in which *A. baumannii* showed high resistance to the most tested antimicrobials. In Campania, the antimicrobial resistance of the *A. baumannii* complex was particularly high, exceeding both the national and European averages. From 2015 to 2019, no significant variations in antimicrobial resistance percentages were observed, with values consistently above the national and European levels. Carbapenem resistance remained above 95% from 2016 to 2018, showing a slight decrease in 2019 (94.7%). Fluoroquinolone resistance ranged between 94% and 97%, peaking in 2018, while gentamicin resistance remained between 93% and 94.6% with no significant variations. Unfortunately, over 90% of isolated strains were simultaneously resistant to three antibiotic classes (carbapenems, fluoroquinolones, and aminoglycosides), indicating that most isolates were MDR, leaving very few therapeutic options [22]. According to the Campania Region’s 2022 Report, 2955 strains of *A. baumannii* were isolated from various biological materials, highlighting high rates of resistance to carbapenems, fluoroquinolones, and aminoglycosides. These data show significantly higher resistance than the national data provided by the AR-ISS report and the European data from the EARS-Net system. Antimicrobial resistance trends remained relatively stable over time; however, more than 80% of isolates were resistant to at least three out of the five antimicrobial classes considered [23].

European surveillance data indicate that *A. baumannii* remains a major challenge due to its high levels of antibiotic resistance. According to the EARS-Net 2019 report [24], between 2015 and 2019, carbapenem resistance in Europe ranged from 31% to 33%, with the highest levels observed in southern and eastern Europe. Resistance to ciprofloxacin was approximately 37%, and resistance to gentamicin remained stable at approximately 31–33%. Combined resistance to carbapenems, fluoroquinolones, and aminoglycosides increased over the period, reflecting the growing frequency of multidrug-resistant (MDR) strains. Italy has consistently seen some of the highest resistance rates in Europe, with carbapenem resistance exceeding 90% and ciprofloxacin resistance approaching 99% by 2019 [25,26]. Colistin, previously used as a last-resort therapy, also showed an emerging resistance trend, increasing from <1% to 2.9% in 2019 [26]. Our retrospective analysis confirms and extends these observations to a regional Italian cohort. Using regression analysis, we quantified temporal trends in resistance for key antibiotics. Consistent with national data, ciprofloxacin resistance showed a significant annual increase (~46% per year), while gentamicin resistance also increased substantially (~71% per year), reflecting its continued use in combination therapies for multidrug-resistant infections. For imipenem, we observed a small, non-significant decrease in resistance, consistent with the modest downward trend reported in the most recent ECDC data from 2019 to 2023 [27]. Resistance to trimethoprim/sulfamethoxazole decreased significantly (~10% per year), suggesting some residual susceptibility that could guide future therapeutic strategies (Table 6).

*A. baumannii* can also be involved in polymicrobial infections, especially in immunocompromised patients admitted to the ICU [28]. The co-infection with other bacteria worsens the patient’s condition, making treatment more challenging and increasing the risk of complications. Our analysis of respiratory samples co-infected with *A. baumannii* revealed that the most frequently co-isolated pathogens were *A. baumannii* were *K. pneumoniae* (*K. pneumoniae)* (28.8%), *S. aureus* (22.2%), and *P. aeruginosa* (14.8%) (Table 9 and Table 10). The temporal trend shows an increase in *K. pneumoniae* and *S. aureus* infections over the years. In agreement with our data, Karakonstantis et al. found that the most common co-isolates in pulmonary infections result to be *P. aeruginosa*, followed by *S. aureus* and *Klebsiella* spp. [29]. Polymicrobial infections involving *K. pneumoniae* and *A. baumannii* are frequently associated with increased severity, prolonged hospital stays, and higher antimicrobial resistance. While these pathogens are often co-isolated in clinical settings, the mechanisms underlying their coexistence and potential synergy remain poorly understood. Semenec et al. demonstrated the reciprocal metabolic and protective interactions between *K. pneumoniae* and *A. baumannii.* enhances the growth of *A. baumannii* by supplying fermentation by-products as alternative carbon sources and producing metabolites such as ethanol and lactate, improving its growth in nutrient-limited environments. At the same time, *A. baumannii* provides *K. pneumoniae* with cross-protection against the cephalosporin antibiotic cefotaxime, to which *K. pneumoniae* is partially susceptible. In mixed cultures, *A. baumannii* reduced the antibiotic’s effectiveness by secreting β-lactamases, allowing *K. pneumoniae* to survive in cefotaxime concentrations that would otherwise be lethal in monoculture conditions. These findings suggest that the interaction between these two bacteria not only facilitates their coexistence in polymicrobial infections but also contributes to increased virulence and antimicrobial resistance, leading to difficult-to-treat infections [30]. On the other hand, Timme et al. showed the *S. aureus* capability to interfere with *A. baumannii* by the expression of phenol-soluble modules. At the same time, by providing acetoin as an alternative carbon source, *S. aureus* promotes *A. baumannii* growth, establishing competitive and cooperative strategies by cross-feeding mechanisms [31].

In a study conducted on 146 patients, 60 patients had a co-infection, with 75% of cases involving co-infection with carbapenem-resistant *K. pneumoniae* (CRKP) and *P. aeruginosa*. Additionally, 13% and 7% of cases involved co-infections of *K. pneumoniae* with *A. baumannii* and co-infections of *K. pneumoniae*, *A. baumannii*, and *P. aeruginosa*, respectively. Polymicrobial infections involving *K. pneumoniae* and *A. baumannii* are associated with increased severity, prolonged hospitalization, and higher antimicrobial resistance. In line with our study, the respiratory tract was the most frequently analyzed site due to its high frequency of co-infections [32]. Managing MDR *A. baumannii* infections in ICUs presents a clinical and microbiological challenge, with significant implications for hospital infection control [33]. 11 May 2025 9:05:00 a.m. In our research, the most frequent co-infections involved *K. pneumoniae* and *S. aureus*. These findings highlight the need for more effective infection control strategies, prudent antibiotic use, and further research on co-infections to improve clinical management [34]. Co-infections with MDR microorganisms complicate clinical management for several reasons, as they increase antimicrobial resistance and render it more difficult to select an effective treatment [35]. Implementing robust antimicrobial stewardship programs and reinforcing infection control protocols remain essential steps in reducing the spread of MDR *A. baumannii* and its role in nosocomial outbreaks [36]. This study has some limitations. Clinical information, such as patient symptoms, antimicrobial therapy, length of hospital stays, and clinical outcomes, was not available for analysis. The dataset was limited to microbiological data (bacterial strain identification), along with basic demographic variables (age and sex), and the hospital department of admission. Consequently, the lack of detailed clinical data limits the ability to correlate microbiological findings with disease severity, therapeutic response, and patient prognosis.

## 5. Conclusions

This study provides a comprehensive overview of *A. baumannii* infections and antimicrobial resistance patterns observed at a tertiary care hospital in southern Italy over nine years (2015–2023). The results confirm the persistence of *A. baumannii* as an important multidrug-resistant pathogen, with particularly high resistance rates to ciprofloxacin, imipenem, and trimethoprim/sulfamethoxazole in both study periods. A statistically significant and progressive increase in resistance was observed for ciprofloxacin and colistin, the latter also showing an alarming increase during the years following COVID-19. In contrast, gentamicin and imipenem showed relatively stable resistance profiles, while trimethoprim/sulfamethoxazole was the only antibiotic to show a modest but significant decrease in resistance over time, indicating a potential, albeit limited, improvement in susceptibility. The increased frequency of *A. baumannii* infections during the pandemic period (2020–2023) may be related to increased intensive care unit admissions, greater use of invasive ventilation, and intensified empiric antibiotic therapy during COVID-19 hospitalizations. These observations are consistent with European reports highlighting pandemic-related changes in antimicrobial resistance dynamics. Overall, the findings underscore the urgent need to strengthen infection prevention and control measures, optimize antibiotic stewardship programs, and promote continuous surveillance to mitigate the spread of *A. baumannii* and preserve the clinical efficacy of last-resort treatments such as colistin.

## Figures and Tables

**Table 1:** Gender distribution of patients by year (2015–2023). The table reports the number of male and female patients for each year, with percentages shown in parentheses. Data are presented for the periods 2015–2019 and 2020–2023, as well as the total across each period. Remaining proportion. Total counts per year are also reported.

Years Gender	2015	2016	2017	2018	2019	2015–2019	2020	2021	2022	2023	2020–2023
Male n. (%)	229 (65.6)	223 (67.6)	200 (57.5)	248 (68.7)	197 (67.7)	1097 (65.3)	198 (73.1)	186 (72.4)	206 (58.2)	174 (57.2)	764 (64.4)
Female n. (%)	120 (34.4)	107 (32.4)	148 (42.5)	113 (31.3)	94 (32.3)	582 (34.7)	73 (26.9)	71 (58,2)	148 (41.8)	130 (42.8)	422 (35.6)
Total	349	330	348	361	291	1679	271	257	354	304	1186

**Table 2:** Frequency of male and female patients per year, divided by age group (2015–2019). The table shows the annual distribution of positive *A. baumannii* infections by gender and age group for the period 2015–2019. Data are expressed as the number of cases, with percentages (in parentheses) representing the proportion within each category. This allows comparison of infection rates between males and females across different age groups and years.

	2015		2016		2017		2018		2019		2015–2019	
Years	M (%)	F (%)	M (%)	F (%)	M (%)	F (%)	M (%)	F (%)	M (%)	F (%)	M (%)	F (%)
0–10	0	0.8	0	0.9	0	0	0	0	0	0	0	0.3
11–20	6.1	4.2	3.1	0	1.5	4.1	2.8	0.9	11.2	0	4.8	2.1
21–30	5.2	1.7	5.4	4.7	5.0	2.7	4.4	1.8	8.1	0	5.6	2.2
31–40	5.2	4.2	4.0	16.8	1.5	2.0	6.0	4.4	2.5	2.1	4.0	5.7
41–50	11.4	7.5	5.8	11.2	13.0	14.2	7.7	6.2	9.1	9.6	9.3	10.0
51–60	14.0	12.5	18.8	15.0	17.5	8.8	19.0	9.7	11.7	11.7	16.3	11.3
61–70	18.8	22.5	25.1	11.2	30.0	16.9	26.6	21.2	19.3	25.5	24.0	19.2
71–80	22.3	25.8	26.0	19.6	18.5	30.4	20.6	37.2	16.8	24.5	21.0	27.8
81–90	16.6	19.2	11.2	18.7	12.0	20.3	11.3	17.7	21.3	24.5	14.3	19.9
91–100	0.4	1.7	0.4	1.9	1.0	0.7	1.6	0.9	0	2.1	0.7	1.4
Total	229	120	223	107	200	148	248	113	197	94	1097	582
*p*-value	0.442		<0.001		0.003		0.02		<0.001		<0.001	

**Table 3:** Frequency of male and female patients per year, divided by age groups (2020–2023). The table reports the annual distribution of *A. baumannii*-positive infections by gender and age group for the period 2020–2023. Data are presented as the number of cases, with percentages (in parentheses) indicating the proportion within each category. This representation allows comparison of infection frequency between males and females across different age groups and years during the post-2019 period.

	2020		2021		2022		2023		2020–2023	
Years	M (%)	F (%)	M (%)	F (%)	M (%)	F (%)	M (%)	F (%)	M (%)	F (%)
0–10	0	0	0	0	0.5	0	0.6	0	0.3	0
11–20	0.5	0	0.5	0	3.4	0	2.3	0	1.7	0
21–30	0.5	4.1	9.1	1.4	0	6.1	1.7	0	2.7	3.1
31–40	10.1	4.1	2.7	2.8	2.9	18.2	4.0	1.5	5.0	8.1
41–50	10.6	8.2	5.4	4.2	7.8	12.2	6.9	1.5	7.7	6.9
51–60	18.7	8.2	16.7	21.1	25.2	7.4	17.8	19.2	19.8	13.5
61–70	29.8	32.9	22.6	31.0	20.9	10.8	26.4	14.6	24.9	19.2
71–80	22.7	28.8	34.9	23.9	21.4	28.4	24.1	28.5	25.7	27.7
81–90	7.1	11.0	8.1	15.5	17.0	16.2	16.1	30.8	12.0	19.7
91–100	0	2.7	0	0	1.0	0.7	0	3.8	0.3	1.9
Total	198	73	186	71	206	148	174	130	764	422
*p*-value	0.02		0.07		<0.001		<0.001		<0.001	

**Table 4:** Distribution of *A. baumannii* isolates from 2015 to 2019 by specimen type and infection site (from highest to lowest). The table summarizes the distribution of *A. baumannii* isolates collected between 2015 and 2019 by specimen type or infection site. Data are presented as the number of isolates (n) and the corresponding percentage (%) for each year and for the entire period 2015–2019.

Years	2015–2019		2015		2016		2017		2018		2019	
Samples	Isolates n.	Isolates %	Isolates n.	Isolates %	Isolates n.	Isolates %	Isolates n.	Isolates %	Isolates n.	Isolates %	Isolates n.	Isolates %
Respiratory	782	46.6	161	46.1	144	43.6	151	43.4	166	46.0	160	55.0
Wound swab	248	14.8	56	16.0	52	15.8	45	12.9	52	14.4	43	14.8
Blood culture	197	11.7	39	11.2	32	9.7	47	13.5	52	14.4	27	9.3
Urinary	183	10.9	34	9.7	38	11.5	43	12.4	40	11.1	28	9.6
Cultural swab	132	7.9	28	8.0	22	6.7	32	9.2	31	8.6	19	6.5
Catheters	93	5.5	25	7.2	25	7.6	18	5.2	20	5.5	5	1.7
Liquor culture	27	1.6	2	0.6	11	3.3	8	2.3	0	0	6	2.1
Vaginal swab	12	0.7	2	0.6	5	1.5	4	1.1	0	0	1	0.3
Others	5	0.3	2	0.6	1	0.3	0	0	0	0	2	0.7
Total	1679	100	349	100	330	100	348	100	361	100	291	100

**Table 5:** Distribution of *A. baumannii* isolates from 2020 to 2023 by specimen type and infection site (from highest to lowest). The table presents the distribution of *A. baumannii* isolates collected between 2020 and 2023, categorized by material or site of infection. Data are expressed as the number of isolates (n) and corresponding percentage (%) for each year and for the overall 2020–2023 period.

Years	2020–2023		2020		2021		2022		2023	
Samples	Isolates n.	Isolates %	Isolates n.	Isolates %	Isolates n.	Isolates %	Isolates n.	Isolates %	Isolates n.	Isolates %
Respiratory	536	45.2	137	50.6	129	50.2	147	41.5	123	40.5
Blood culture	178	15.0	60	22.1	43	16.7	47	13.3	28	9.2
Urinary	171	14.4	27	10.0	24	9.3	57	16.1	63	20.7
Cultural swab	127	10.7	20	7.4	21	8.2	49	13.8	37	12.2
Wound swab	115	9.7	14	5.2	27	10.5	35	9.9	39	12.8
Catheters	36	3.0	4	1.5	12	4.7	11	3.1	9	3.0
Vaginal swab	10	0.8	1	0.4	1	0.4	7	2.0	1	0.3
Liquor culture	8	0.7	4	1.5	0	0	0	0	4	1.3
Others	5	0.4	4	1.5	0	0	1	0.3	0	0
Total	1186	100	271	100	257	100	354	100	304	100

**Table 6:** Statistical adjustment for multiple comparisons in antimicrobial resistance trend analysis. Odds ratios (OR) and 95% confidence intervals (CI) are reported for each antibiotic. To account for multiple simultaneous tests, *p*-values were adjusted using the Benjamini–Hochberg (BH) method to control the false discovery rate (FDR). The table indicates whether the association between antibiotic resistance and the analyzed factor is statistically significant after BH adjustment.

Antimicrobials	Odds-Ratio	IC 95%	BH-Adjusted *p*-Value	Significant
Ciprofloxacin	1.63	1.27–1.63	2.99 × 10^−8^	TRUE
Colistin	1.82	1.43–1.82	3.11 × 10^−14^	TRUE
Gentamicin	0.45	0.93–1.03	0.445	FALSE
Imipenem	0.17	0.90–1.01	0.167	FALSE
Trimetoprim/Sulfam.	2.52	0.89–0.98	0.0252	TRUE

**Table 7:** Antibiotic resistance profiles of *A. baumannii* (%) with *p*-values and trend analysis for the period 2015–2019. The table summarizes the annual resistance rates of *A. baumannii* isolates to selected antibiotics from 2015 to 2019. Data are expressed as the number of isolates tested (n) and the corresponding percentage of resistant strains (R%). Statistical significance (*p*-value) was calculated to assess differences in resistance rates across years, while trend analysis (*p*-value trend) was performed to assess temporal variations over the study period.

Antimicrobials	2015		2016		2017		2018		2019		2015–2019		*p*-Value	*p*-Value Trend
	n.	R%	n.	R%	n.	R%	n.	R%	n.	R%	n.	R%		
Ciprofloxacin	349	96.85	328	93.90	348	96.84	361	96.12	291	99.31	1677	96.54	0.007	0.04
Colistin	340	0	311	0.64	329	0.61	325	0.92	273	2.90	1578	0.95	0.004	<0.001
Imipenem	346	96.24	327	92.66	348	96.26	313	95.53	34	94.12	1368	95.18	0.17	0.89
Trim./Sulfam.	349	93.98	327	91.44	348	95.69	361	94.46	291	90.72	1676	93.38	0.06	0.51
Gentamicin	349	92.8	328	90.2	348	87.1	361	92.2	291	93.1	1677	91.0	0.03	0.7

Trim./Sulfam: trimethoprim/sulfamethoxazole; n.: isolates number.

**Table 8:** *A. baumannii* antibiotic resistance profiles (%) with *p*-values and trend analysis for the period 2020–2023. The table summarizes the resistance rates of *A. baumannii* isolates to selected antibiotics during the years 2020–2023. Data are expressed as the number of tested isolates (n) and the corresponding percentage of resistant strains (R%). The *p*-value indicates statistical significance among annual resistance rates, while the *p*-value trend reflects the direction and significance of resistance changes over time.

Antimicrobials	2020		2021		2022		2023		2020–2023		*p*-Value	*p*-Value Trend
	n.	R%	n.	R%	n.	R%	n.	R%	n.	R%		
Ciprofloxacin	270	98.1	256	100.0	354	100.0	303	100.0	1183	99.6	<0.001	0.001
Colistin	237	2.5	221	2.3	334	8.4	251	10.0	1043	6.1	<0.001	<0.001
Imipenem	231	95.2	257	92.2	354	94.6	302	93.0	1144	93.8	0.45	0.58
Trim./Sulfam.	270	93.7	257	88.7	355	91.0	277	91.0	1159	91.1	0.25	0.44
Gentamicin	270	93.7	256	90.6	355	89.3	305	89.2	1186	90.6	0.21	0.05

Trim./Sulfam: trimethoprim/sulfamethoxazole; n.: isolates number.

**Table 9:** Distribution of bacterial species co-isolated with *A. baumannii* in respiratory samples (2015–2019). The table shows the number (n) and percentage (%) of isolates of different bacterial species identified as co-infected with *A. baumannii* in respiratory samples. Data are presented for each year from 2015 to 2019, as well as aggregated for the entire period 2015–2019. Species are ranked from highest to lowest frequency. “Others” includes bacterial species with lower frequencies not listed individually.

Years	2015–2019	2015	2016	2017	2018	2019
Bacterial Species in Co-Infection	Isolates n. (%)	Isolates n. (%)	Isolates n. (%)	Isolates n. (%)	Isolates n. (%)	Isolates n. (%)
*Klebsiella pneumoniae*	109 (28.8)	29 (29.3)	34 (45.9)	20 (25.3)	14 (17.5)	12 (26.1)
*Staphylococcus aureus*	84 (22.2)	20 (20.2)	10 (13.5)	22 (27.8)	18 (22.5)	14 (30.4)
*Pseudomonas aeruginosa*	56 (14.8)	16 (16.2)	10 (13.5)	9 (11.4)	15 (18.8)	6 (13.0)
*Proteus mirabilis*	32 (8.5)	3 (3.0)	3 (4.1)	8 (10.1)	12 (15.0)	6 (13.0)
Others	28 (7.4)	8 (8.1)	7 (9.5)	8 (10.1)	4 (5.0)	1 (2.2)
*Escherichia coli*	23 (6.1)	2 (2.0)	6 (8.1)	4 (5.1)	6 (7.5)	5 (10.9)
*Providencia stuartii*	20 (5.3)	10 (10.1)	1 (1.4)	5 (6.3)	3 (3.8)	1 (2.2)
*Enterococcus faecalis*	8 (2.1)	5 (5.1)	1 (1.4)	0 (0)	1 (1.3)	1 (2.2)
*Enterobacter cloacae*	7 (1.9)	0 (0)	1 (1.4)	1 (1.3)	5 (6.3)	0 (0)
*Stenotrophomonas maltophilia*	6 (1.6)	2 (2.0)	0 (0)	2 (2.5)	2 (2.5)	0 (0)
*Candida albicans*	5 (1.3)	4 (4.0)	1 (1.4)	0 (0)	0 (0)	0 (0)
Total	378	99	74	79	80	46

**Table 10:** Distribution of bacterial species co-isolated with *A. baumannii* in respiratory samples (2020–2023). The table presents the number (n) and percentage (%) of isolates of different bacterial species identified in co-infection with *A. baumannii* in respiratory samples from 2020 to 2023. Species are ranked from highest to lowest frequency for each year and for the overall 2020–2023 period. “Others” includes species with low frequencies not listed individually.

Years	2020–2023	2020	2021	2022	2023
Bacterial Species in Co-Infection	Isolates n. (%)	Isolates n. (%)	Isolates n. (%)	Isolates n. (%)	Isolates n. (%)
*Staphylococcus aureus*	67 (28.6)	24 (50)	21 (26.9)	9 (16.7)	13 (24.1)
*Pseudomonas aeruginosa*	43 (18.4)	6 (12.5)	17 (21.8)	11 (20.4)	9 (16.7)
*Klebsiella pneumoniae*	35 (15.0)	10 (20.8)	8 (10.3)	11 (20.4)	6 (11.1)
Others	21 (9.0)	2 (4.2)	11 (14.1)	5 (9.3)	3 (5.6)
*Providencia stuartii*	13 (5.6)	0 (0)	3 (3.8)	3 (5.6)	7 (13)
*Escherichia coli*	10 (4.3)	1 (2.1)	1 (1.3)	2 (3.7)	6 (11.1)
*Proteus mirabilis*	10 (4.3)	1 (2.1)	2 (2.6)	4 (7.4)	3 (5.6)
*Staphylococcus haemolyticus*	10 (4.3)	0 (0)	4 (5.1)	3 (5.6)	3 (5.6)
*Staphylococcus epidermidis*	10 (4.3)	0 (0)	7 (9)	2 (3.7)	1 (1.9)
*Enterococcus faecalis*	9 (3.8)	3 (6.3)	4 (5.1)	1 (1.9)	1 (1.9)
*Enterococcus faecium*	6 (2.6)	1 (2.1)	0 (0)	3 (5.6)	2 (3.7)
Total	234	48	78	54	54

Others: Pseudomonas fluorescens, Staphylococcus epidermidis, Enterobacter aerogenes, Klebsiella oxytoca, Staphylococcus haemolyticus, Citrobacter freundii, Candida tropicalis, Enterobacter asburiae, Hafnia alvei, Raoultella planticola, Serratia marcescens, Staphylococcus simulans, Streptococcus agalactiae, Streptococcus dysgalactiae, and members of the Streptococcus viridans group were collectively categorized as “others” due to their low frequency of detection across the study period.

## Data Availability

The data are contained within the article.

## Supplementary Materials

The following supporting information can be downloaded at: https://www.mdpi.com/article/10.3390/pathogens14111165/s1, Table S1: Distribution by required hospital units and relative frequency (from highest to lowest).
