# Supplementary material for: A Nine-Year Review of Acinetobacter baumannii Infections Frequency and Antimicrobial Resistance in a Single-Center Study in Salerno, Italy
Source: Pathogens. 2025 Nov 14;14(11):1165. doi: 10.3390/pathogens14111165 (PMC12655280; doi:10.3390/pathogens14111165)
Supplement: Supplementary file 1 [file pathogens-14-01165-s001.zip › pathogens-3947601-supplementary.pdf]

Supplementay Table S1. *Distribution by units with relative prevalence (from highest to lowest).*

Units	2015-2019 (%)	Units	2020-2023 (%)
Intensive Care	658 (39.19)	Intensive Care	433 (36.51)
Pulmonology	112 (6.67)	General Medicine	116 (9.78)
Emergency Medicine	104 (6.19)	Critical Care and Anesthesiology	89 (7.50)
Emergency Surgery	99 (5.90)	Infectious Diseases	76 (6.41)
Neurosurgery	66 (3.93)	Pulmonology	69 (5.82)
Critical Care and Anesthesiology	60 (3.57)	Neurosurgery	51 (4.30)
Cardiac Surgery Intensive Care	53 (3.16)	Emergency Surgery	46 (3.88)
Infectious Diseases	52 (3.10)	Cardiac Surgery Intensive Care	40 (3.37)
Orthopedics and Traumatology	50 (2.98)	Emergency Medicine	36 (3.04)
Nephrology	46 (2.74)	Internal Medicine and Hepatology	32 (2.70)
General Medicine (Female)	44 (2.62)	Neurology	26 (2.19)
Outpatient Clinic (Ruggi)	42 (2.50)	Nephrology	22 (1.85)
General Medicine (Male)	40 (2.38)	Emergency Cardiac Surgery	19 (1.60)
Vascular Surgery	33 (1.97)	Outpatient Clinic	16 (1.35)
Hematology	30 (1.79)	General Surgery	13 (1.10)
Coronary Care Unit (CCU)	27 (1.61)	Hematology	11 (0.93)
Emergency Cardiac Surgery	19 (1.13)	Orthopedics and Traumatology	11 (0.93)
Nephrology Day Hospital	18 (1.07)	Coronary Care Unit (CCU)	10 (0.84)
General Medicine	15 (0.89)	Vascular Surgery	8 (0.67)
Neurology	15 (0.89)	General Medicine - Men	8 (0.67)
General Surgery	13 (0.77)	Stroke Unit	7 (0.59)
Cardiology	11 (0.66)	Musculoskeletal Surgery	7 (0.59)
Stroke Unit	9 (0.54)	Cardiology	6 (0.51)
Kidney Transplant and Related Surgery Center	7 (0.42)	General Medicine - Women	5 (0.42)
Oncology Ward	7 (0.42)	Nephrology Day Hospital	5 (0.42)
Functional Recovery and Rehabilitation	7 (0.42)	Maxillofacial Surgery	4 (0.34)
Locomotor System Surgery	6 (0.36)	Functional Recovery and Rehabilitation	4 (0.34)
Hematology Day Hospital	6 (0.36)	Oncology	3 (0.25)
Vascular Surgery Sub-Intensive Care	5 (0.30)	Urology	3 (0.25)
Maxillofacial Surgery	4 (0.24)	High-Risk Pregnancy and Prenatal Diagnosis	3 (0.25)
Psychiatry	4 (0.24)	Obstetrics and Gynecology	2 (0.17)
Medical-Surgical Short Stay Unit (OBI)	3 (0.18)	Psychiatry	2 (0.17)
Orthopedics and Traumatology	3 (0.18)	Otorhinolaryngology (ENT)	1 (0.08)

Gastroenterology	2 (0.12)	Hematology Day Hospital	1 (0.08)
Neonatology	2 (0.12)	Neonatology	1 (0.08)
Obstetrics and Gynecology	2 (0.12)	Kidney Transplant Center and Related Surgery	0 (0)
Urology	2 (0.12)	Vascular Surgery - Sub-Intensive Care	0 (0)
Dialysis Day Hospital	1 (0.06)	Dialysis Day Hospital	0 (0)
High-Risk Pregnancy and Prenatal Diagnosis	1 (0.06)	Gastroenterology (University Unit)	0 (0)
Otolaryngology	1 (0.06)	Medical-Surgical Emergency Observation Unit	0 (0)
Total	**1,679 (100)**	Orthopedics and Traumatology Day Hospital	0 (0)
		Total	**1,186 (100)**
